# Directional and reoccurring sequence change in zoonotic RNA virus genomes visualized by time-series word count

**DOI:** 10.1038/srep36197

**Published:** 2016-11-03

**Authors:** Yoshiko Wada, Kennosuke Wada, Yuki Iwasaki, Shigehiko Kanaya, Toshimichi Ikemura

**Affiliations:** 1 Department of Bioscience, Nagahama Institute of Bio-Science and Technology, Tamura-cho 1266, Nagahama-shi, Shiga-ken 526-0829, Japan; 2 Nara Institute of Science and Technology, 8916-5 Takayama, Ikoma, Nara 630-0192 Japan

## Abstract

Ebolavirus, MERS coronavirus and influenza virus are zoonotic RNA viruses, which mutate very rapidly. Viral growth depends on many host factors, but human cells may not provide the ideal growth conditions for viruses invading from nonhuman hosts. The present time-series analyses of short and long oligonucleotide compositions in these genomes showed directional changes in their composition after invasion from a nonhuman host, which are thought to recur after future invasions. In the recent West Africa Ebola outbreak, directional time-series changes in a wide range of oligonucleotides were observed in common for three geographic areas, and the directional changes were observed also for the recent MERS coronavirus epidemics starting in the Middle East. In addition, common directional changes in human influenza A viruses were observed for three subtypes, whose epidemics started independently. Long oligonucleotides that showed an evident directional change observed in common for the three subtypes corresponded to some of influenza A siRNAs, whose activities have been experimentally proven. Predicting directional and reoccurring changes in oligonucleotide composition should become important for designing diagnostic RT-PCR primers and therapeutic oligonucleotides with long effectiveness.

Viruses have always posed significant threats to public health, as highlighted by the recent ebolavirus outbreak in West Africa^1^,^2^,^3^,^4^ and the emerging and re-emerging nature of influenza viruses^5^. To face the world-wide threats caused by zoonotic RNA viruses, which suddenly cause serious outbreaks by invasion from nonhuman hosts and mutate rapidly, we must understand the molecular evolutionary changes in their genome sequences extensively by innovating various technologies, including those used in big data analyses, e.g., large-scale word count. Time-series word count of oligonucleotides in genome sequences can be conducted without specialized assumptions and prior knowledge and is useful for unsupervised data mining. Importantly the obtained results are easily understandable, even for those unfamiliar with molecular evolutionary studies.

Oligonucleotide composition varies significantly among species even with the same genome G+C%; therefore, the composition, especially of short oligonucleotides, has been used as a phylogenetic parameter “genome signature”^6^ to classify microbial genomes, including viral genomes. Viruses are inevitably dependent on many host factors for their growth (e.g., nucleotide pools, proteins and RNAs) and must escape from host antiviral mechanisms (e.g., interferon-induced systems)^7^,^8^,^9^. Therefore, after the invasion of zoonotic viruses from nonhumans, a certain level of directional change in genomic sequences and thus in mono- and oligonucleotide compositions should occur during human-to-human transmission. In fact, the G+C% of influenza A virus strains isolated from humans is lower than that of strains isolated from natural hosts, which were avian^10^, and the CG dinucleotide in an A/U context has been preferentially eliminated from classical H1N1 influenza viruses during human-to-human transmission^11^. By using BLSOM (Batch Learning Self-Organizing Map)^12^,^13^, we previously found directional compositional changes in a wide range of oligonucleotides^14^,^15^ that occurred after the onset of the influenza H1N1/09 pandemic of 2009^16^,^17^,^18^.

In this study, we first conducted time-series word counts on changes in mono- to pentanucleotide compositions, occurring after invasion from nonhumans, for ebolavirus, MERS coronavirus and influenza viruses. Then, as an example of long oligonucleotides, we analyzed 20-mers. Because 20-mers are composed of 4^20^ (*ca.* 1.1 trillion) variables, the analysis becomes a big data analysis. Long oligonucleotides are key elements of diagnostic PCR primers^19^ and potentially promising nucleic-acid therapeutics (e.g., antisense RNA, siRNA and miRNA)^19^,^20^,^21^,^22^,^23^. RNA viruses mutate very rapidly, and therefore, their sequence changes should be intensively characterized for designing diagnostic and therapeutic oligonucleotides with long effectiveness. The present time-series analysis on influenza A genomes showed that the 20-mers exhibiting the most evident directional changes observed in common for three different subtypes corresponded to several influenza A siRNAs, which were experimentally validated. Time-series analyses can provide information on changes possibly reoccurring after invasions from natural reservoir hosts.

## Results and Discussion

### Spatiotemporal analysis of mononucleotide composition in ebolavirus genomes

Because of the recent major threat to public health in West Africa, more than 1000 genomes of Zaire ebolavirus (EBOV) strains, isolated from humans from 2014 to 2015, have been sequenced, and the sequences are available from the NCBI Virus Variation Database^24^ (http://www.ncbi.nlm.nih.gov/genome/viruses/variation/ebola/). To study molecular evolutionary changes in EBOV genomes in the current epidemic, we obtained 1020 strains, for which the isolated dates are given. Although we focused on full-length genomes, a minor portion was relatively short in length, or many unidentified “N” bases were included, partly because of the stringent sterilization treatment obligated by the local government^3^. Because these will give odd values of mono- and oligonucleotide compositions, we focused on 935 genomes longer than 18.5 kb after omitting N bases, which were derived from 244 Guinea, 156 Liberia and 535 Sierra Leone strains. To conduct the present spatiotemporal analysis, 15 strains isolated from several other geographic areas were omitted.

**Figure 1:**
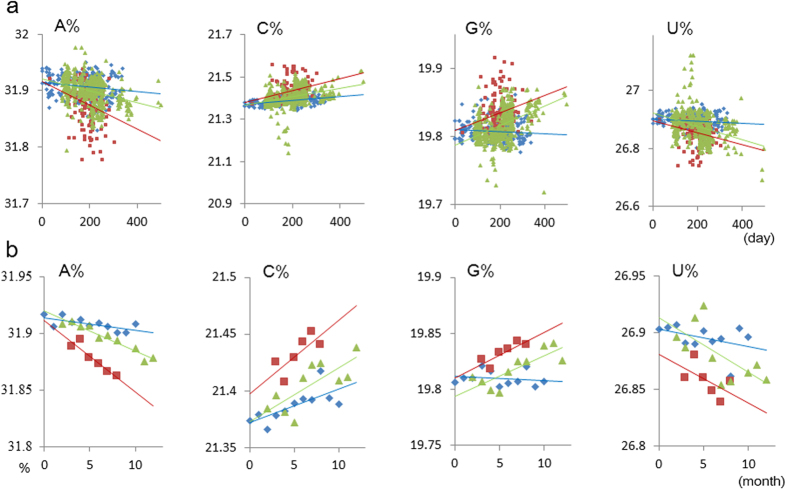
Time-series changes in mononucleotide composition (%) for EBOV. (**a**) Mononucleotide (A, C, G and U) composition for each strain is plotted according to the elapsed days from Mar. 17, 2014, the first day for virus isolation noted by the Ebolavirus Database^24^. Data and regression lines separately analyzed for different geographic areas are differentially colored: Guinea (blue), Liberia (brown) and Sierra Leone (green). (**b**) Averaged mononucleotide compositions for strains in each month are plotted according to the elapsed months from March 2014. Data and regression lines for the elapsed months are colored as described in (**a**). A separate and additional regression analysis for data without the averaging (**a**) that can examine time-series changes from the epidemic start for all three areas showed that the null hypothesis (i.e. no correlation) was rejected for all mononucleotides in the three areas (12 cases) at the significance level of 0.01, except for T% and G% for Guinea; this was rejected for the T% for Guinea at the significance level of 0.05. For data after averaging (**b**), the null hypothesis was rejected at the significance level of 0.05 for mononucleotides in the three areas, except for G% and T% in Guinea.

We first analyzed time-series change in mononucleotide composition for strains isolated in the three areas separately. Figure 1a plots four mononucleotide compositions (%) in each genome according to the isolated day, which started on March 17, 2014. Because mutations occur primarily as random processes, mononucleotide compositions for strains isolated even around the same day clearly differed from each other, which resulted in evident compositional diversity. Despite this diversity, which should also be due to sequencing uncertainty, linear regression lines appear to show a time-dependent increasing or decreasing trend, a possible increase for C% and G% and a possible decrease for A% and U%. This is consistent with the excess U-to-C mutations found by two groups^2^,^3^.

The present study focused on time-series changes, but not on data variations caused largely by random mutations and sequencing uncertainty, and therefore, average characteristics of strains isolated in a similar period were analyzed, summing up the sequences isolated in one month and calculating an averaged mononucleotide composition for each month (Fig. 1b). This could be done because the genome sequence data were distributed rather evenly by area and month; the average number of strains per month was 22.2, 41.2 and 17.3 for Guinea (blue), Sierra Leone (green) and Liberia (brown), respectively. We omitted the data for a few months, for which no more than 5 strains were available in the respective area.

Figure 1b shows a more conspicuous increasing or decreasing trend than Fig. 1a, and the time-series increasing or decreasing trend revealed by the regression line for the elapsed months was the same among the three areas for all nucleotides, except for an ambiguous case of G% for Guinea. In more detail, positive and negative directions of the regression line were primarily common among the three areas, although the slope angle appear to differ; Table 1 lists Pearson’s correlation coefficients calculated separately for the three areas. The highest negative (−0.94) or positive (0.92) correlation among mononucleotides was observed for A% or C%, respectively, in Liberia, which corresponded to a decrease of 10.2 As or an increase of 11.4 Cs per genome at the final stage of the current outbreak. Notably, the increasing or decreasing trend detected by these averaged data for one month (Fig. 1b) is common to the result without averaging (Fig. 1a).

In these regression analyses, the strains that were isolated in the three areas were independently analyzed. A separate regression analysis that could examine the statistical significance of directional changes from the epidemic start for all three areas, was conducted, by including the data of strains isolated at or near the beginning of the current epidemic (i.e., 2014 March strains in Guinea) in Liberia and Sierra Leone data. The results thus obtained by the latter analysis were consistent with those obtained by the former, and the null hypothesis (i.e. no correlation) was rejected at statistically significant levels in most cases, as described in Figure legend in detail.

### Short oligonucleotide composition in ebolavirus genomes

**Figure 2:**
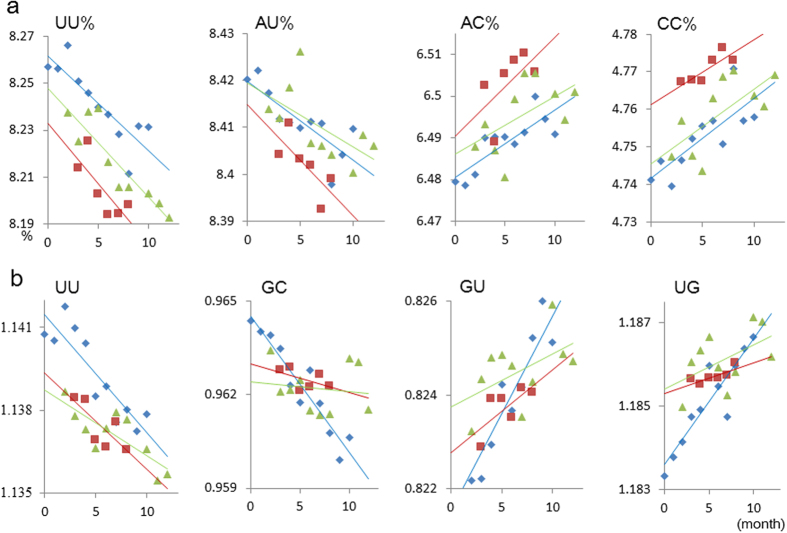
Time-series changes in dinucleotide compositions (%) for EBOV. (**a**) Averaged dinucleotide compositions for strains in each month and regression line are presented in the color distinguished geographic areas, as described in Fig. 1. Four dinucleotides with decreasing or increasing trends are arranged primarily according to the absolute value of their averaged correlation coefficient for the three areas. The separate regression analysis explained in the legend for Fig. 1 showed that the null hypothesis (i.e. no correlation) was rejected for all four dinucleotides in the three areas at the significance level of 0.05; for 10 of the 12 cases, this was rejected even at the significance level of 0.01. (**b**) Ratio of the observed to the expected occurrence (obs/exp) is presented for four dinucleotides and other examples are presented in Supplementary Fig. 1aii. The null hypothesis was rejected for all cases, except for GC in Sierra Leone, at the significance level of 0.02.

Next, we analyzed each dinucleotide composition in the genomes grouped for each month. The results presented in Fig. 2a, Table 1 and Supplementary Fig. 1a show that a common increasing or decreasing trend was observed among the three areas for more than half of the sixteen dinucleotides. Correlation coefficients higher than 0.8 or less than −0.8 are **colored** in Table 1. For nine of the ten **colored** dinucleotides, the coefficient in the other two areas primarily shows the common directional trend. These high correlation coefficients should reflect the evolutionary dependence of the genome sequences.

A decrease in UU and AU and an increase in CC (Fig. 2a) are predictable, even as a cumulative effect of their constituent mononucleotides (Fig. 1b), but an increase in AC, CU, GU and UG is not predictable as a simple cumulative effect and may indicate the characteristics of the contiguous sequence itself. We thus calculated the ratio of the observed dinucleotide occurrence to that expected from the mononucleotide composition (obs/exp) and found a decrease for UU and GC and an increase for GU, UG, AC and UA, which was common among the three areas (Fig. 2b and Supplementary Fig. 1a). The slope direction of the regression line was common among the three areas, but the slope angle appeared to differ among the areas for a wide range of dinucleotides, as well as for tri- and tetranucleotides (data not shown).

A spatiotemporal word count can be conducted without specific assumptions or prior knowledge and visualizes viral evolutionary changes in an easily understandable way. Two distinct models can explain the common increasing or decreasing trend among the three areas, which was observed for a wide range of mono- and oligonucleotides. [Model 1] Throughout the current EBOV epidemic, virus movement between the three areas was extensive; therefore, the time-dependent trend of directional changes in oligonucleotide composition was common among the areas. [Model 2] Even without the extensive mixing of viruses, common directional changes can occur. For example, viruses inevitably depend on many host factors for their growth, but human cells may not provide an ideal growth environment for invading viruses; therefore, common directional changes occur after invasions from nonhumans for supporting more efficient growth in human cells. In this connection, Park *et al.*^3^ reported there was no clear evidence for import or export of EBOV across national borders after its initial introduction. Furthermore, although an increasing or decreasing trend for a wide range of mono- and oligonucleotides is common among the three areas, their actual composition (%) and the slope angle of the directional change often differ among the areas. This observation is consistent with Model 2 and the report by Park *et al.*^3^ but not with Model 1, as discussed below from various aspects.

When considering the cellular environment differences between natural hosts (bats) and humans, not only the low-molecular substances (e.g., nucleotide pool) but also the macromolecules (e.g., proteins) involved in viral growth must be considered. Additionally, antiviral mechanisms^7^,^8^,^9^,^10^ should differ between humans and natural hosts. Host macromolecules involved in viral growth (either supporting or inhibiting) should primarily recognize oligonucleotides, such as several nucleotides or longer, rather than mononucleotides. Hence, we analyzed time-series changes in pentanucleotide (5-mer) occurrences. We first calculated correlation coefficients for all 1024 (=4^5^) 5-mers and sorted them by the averaged correlation coefficients for the three areas.

**Figure 3:**
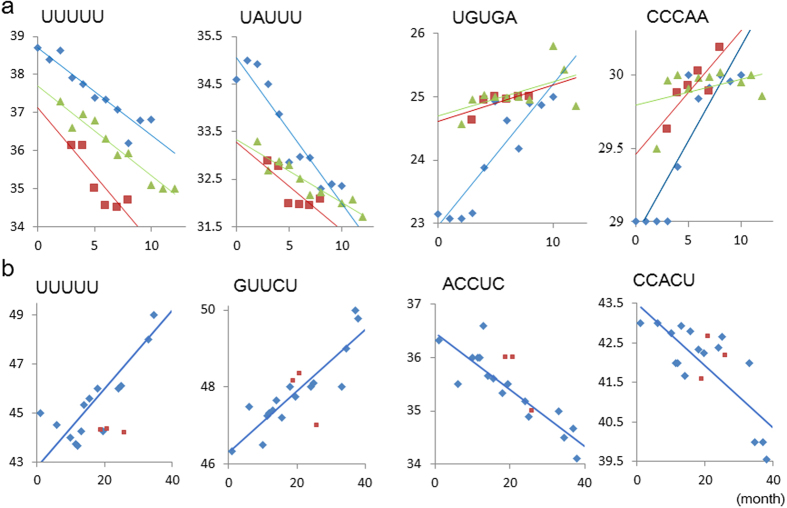
Time-series change in 5-mer occurrence per genome. (**a**) Averaged 5-mer occurrence for EBOV strains in each month and regression lines are colored as described in Fig. 1. Four additional examples are presented in Supplementary Fig. 1b. The null hypothesis was rejected for all 12 cases at the significance level of 0.05; for 11 cases, this was rejected even at the significance level of 0.02. (**b**) Averaged 5-mer occurrences for MERS viruses in each month are plotted according to the elapsed months from April 2012. Camel-derived strains are specified by small brown symbols, and regression lines (blue) were calculated only for human-derived strains. The null hypothesis was rejected for all four 5-mers at the significance level of 0.01, with or without camel-derived strains.

Figure 3a and Supplementary Fig. 1b show time-series patterns of 5-mers with evidently high or low correlation coefficients; 384 or 50 (of 1024 types) 5-mers showed a common decreasing or increasing trend among the areas, respectively. Examination of the ratio of the observed occurrences to those expected from the mononucleotide composition (obs/exp) showed that directional changes of many 5-mers cannot be explained by a cumulative effect of mononucleotide changes (data not shown), as found for various dinucleotides. When further examining the 5-mers showing the common directional change in more detail, their actual occurrence level and slope of the regression line often differed among the areas (Fig. 3a and Supplementary Fig. 1b), and the difference often became clearer compared to that for mono- and dinucleotides. This cannot be explained by the aforementioned Model 1. If the time-series directional change in mono- and oligonucleotide composition found for EBOV is actually due to the necessity of supporting efficient growth in human cells (Model 2), such directional changes should be observed for other zoonotic RNA viruses.

### Directional change in MERS virus genomes

We next analyzed MERS coronaviruses isolated in the recent epidemic starting in April 2012 in the Middle East^25^,^26^. The EBOV outbreak analyzed above was initiated by a single virus strain being introduced from a nonhuman animal; this virus was then transmitted among humans during the outbreak. In contrast, MERS viruses were repeatedly transmitted from nonhumans, mainly camels, in this epidemic^25^, although its original natural host is thought to be bats^26^. Because direct nonhuman hosts for different human isolates differ among isolates, information on camel-derived strains in the current epidemic is important. From the NCBI Virus Variation Database^24^, we found 91 and 16 strains from humans and camels, respectively, with known isolated dates. The total number of MERS virus genomes is approximately one tenth of EBOV genomes, but their sequence length, even after omitting N bases, is mostly similar to its genome size (ca. 30 kb); only one sequence that was evidently short (29.3 kb) was omitted from this analysis. We grouped sequences in each month and analyzed the averaged mono- and oligonucleotides (from 2 to 5-mers) for each month and host.

**Figure 4:**
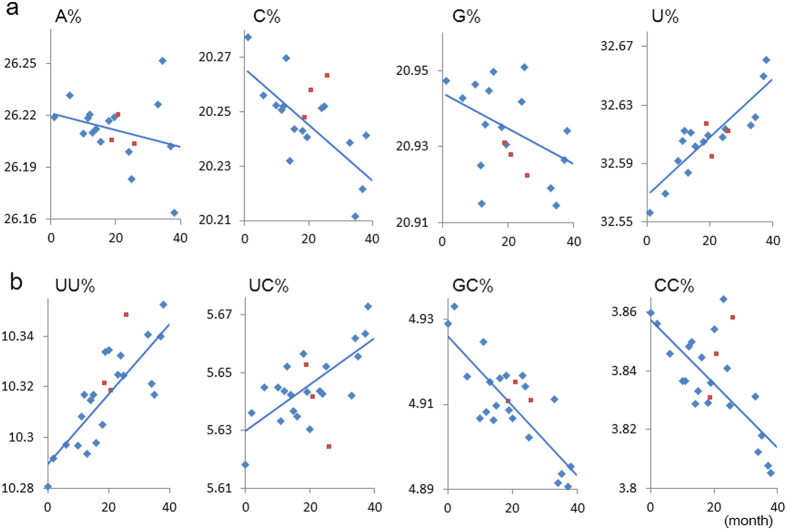
Time-series changes in mono- and dinucleotide composition (%) for MERS viruses. Averaged mono- and dinucleotide compositions (**a,b)**, for strains in each month are plotted according to the elapsed months from April 2012. Camel-derived strains are specified by small brown symbols, and regression lines (blue) were calculated only for human-derived strains. The null hypothesis was rejected for all data (8 cases) at the significance level of 0.01, except for A% and G%, with or without camel-derived strains; this was rejected for the G% at the significance level of 0.1.

Clear time-dependent increasing or decreasing trends were observed for a wide range of mono- and dinucleotides (Fig. 4a,b), but the increasing or decreasing trend found for actual mono- and oligonucleotides clearly differed from EBOV; e.g., C% and G% decrease and U% increases in MERS viruses. EBOV is a negative-sense single-stranded RNA virus, and the sequences registered in the database and analyzed here were complementary to viral genome sequences, but MERS virus is a positive-sense single-stranded RNA virus and, therefore, genome sequences were analyzed. This difference, however, cannot explain differences in directional changes in mono- and oligonucleotide composition of EBOV and MERS viruses.

Figure 3b presents four 5-mers with a high increasing or decreasing trend; e.g., during this 38-month epidemic, approximately six UUUUUs or four CCACUs have been gained or lost per genome, respectively. The time-dependent gain of U_5_ (Fig. 3b) clearly differs from its loss for EBOV (Fig. 3a). Although the regression lines (blue lines in Figs 3b and 4) were calculated only for human strains, occurrence data for camel strains (brown) were primarily positioned around the regression line, thus fitting the previous report that MERS viruses were repeatedly transmitted between camels and humans in the present epidemic^25^. Because time-series directional changes in mono- and oligonucleotide compositions were observed for both EBOV and MERS viruses, the directional change should occur for other zoonotic RNA viruses.

### Mononucleotide composition in influenza virus genomes

We next analyzed influenza viruses, for which a large number of sequences are available from the Influenza Research Database^27^ for different human A subtypes, epidemics of which have started independently at long intervals, such as several decades. Importantly, the aforementioned Model 2, in which the common directional change between different viral populations is expected without extensive virus mixing, is testable more rigorously than for the EBOV outbreak, which started from a single invasion in Guinea and expanded to other countries. Furthermore, we may examine the following important issue. If a common directional change is observed, what types of changes likely reoccur in future epidemics caused by invasions from natural reservoir hosts?

In addition to human A subtypes, genome sequences of human B type, which can currently infect humans but not birds, and of various avian A subtypes were available. The genomes of influenza A and B are composed of eight segments, and we first selected *ca.* 25,000 strains with a full set of eight sequences, categorized the full genome sequences according to host and serological type, and grouped them in each year. The sequence length cut-off was not included because the genome is composed of eight segments of various lengths and a simple, satisfactory criterion for length cut-off could not be set. Alternatively, because 20 times more sequences are available than for EBOV, we selected years with at least ten strains of one category to reduce the artefactual effects derived from sequencing uncertainty. Then, to analyze time-series changes, we selected human or avian A subtypes with more than five years fulfilling the above threshold (≥ten strains per year); the human B type also fitted these criteria. In the case of H1N1/09^16^,^17^,^18^ (starting from 2009 and abbreviated pH1N1 in the database), a sufficient number of sequences fulfilling the above threshold was available even per month and, therefore, pH1N1 sequences in each month were analyzed; importantly, this allowed the study of changes occurring within one outbreak in detail. Collectively, we focused on thirteen subtypes (three human and nine avian A subtypes and a human B type), and first analyzed time-series changes in mononucleotide compositions.

**Figure 5:**
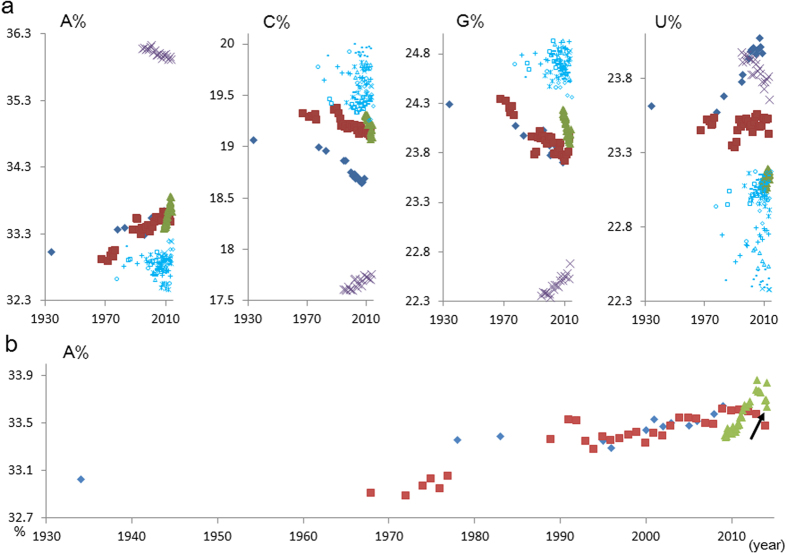
Time-series changes in mononucleotide composition (%) for influenza viruses. (**a**) Averaged mononucleotide compositions for strains in each year are plotted according to the elapsed years from 1930, for human **H1N1** and **H3N2** A subtypes and human **B** type, as well as nine **avian A** subtypes. In the case of **pH1N1**, the averaged compositions for strains in each month are plotted according to the elapsed months from March 2009. Correlation coefficient for human H1N1, H3N2 and pH1N1: 0.91, 0.93 and 0.92 for A%, −0.87, −0.81 and −0.88 for C%, −0.91, −0.88 and −0.92 for G%, and 0.81, 0.16 and 0.69 for U%, respectively. The null hypothesis was rejected for all four mononucleotides in three subtypes (12 cases) at the significance level of 0.01, except for U% for H3N2. Seven **avian A** subtypes are marked separately; H1N1 (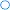), H3N2 (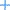), H3N8 (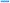), H4N6 (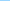), H5N1(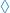), H5N2 (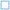), H6N2 (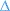), H7N3 (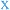) and H7N9 (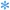). The same colored and smaller symbols than for human subtypes are used for avian A subtypes because differences among avian subtypes were not a concern in the present study. Because the B type has moved gradually toward human A subtypes, this type appears not to have reached its hypothesized goal, which may possibly lie between human A and B types, but nearer the latter. (**b**) A horizontally long panel for A% of three human A subtypes is presented for clarifying detailed changes occurring within and after one outbreak of pH1N1. Data of C% and G% are presented in Supplementary Fig. 2a.

Three human subtypes of influenza A virus (human H1N1, H3N2 and pH1N1) changed their A%, C% and G% with common increasing or decreasing trends among the three subtypes (Fig. 5a), despite different epidemics having started by independent invasions from nonhumans; the G+C% increase in human A subtypes was previously reported by Rabadan *et al.*^10^. Figure 5a shows that the A%, C% and G% move time-dependently apart from those of avian A subtypes (sky blue) and toward those of the human B type (violet). Because the B type can currently infect humans, but not birds, this type has possibly adapted better to growth in human cells than A subtypes. The directional change of human A subtypes “apart from avian A subtypes and toward the human B type” may visualize their evolutionary journey from each start of human-to-human transmission. In the case of U%, human H1N1 (blue) and H3N2 (brown) have already reached the B’s level, and only pH1N1 (green) shows an increasing trend toward B.

A large number of pH1N1 strains isolated from 2009 to 2014 could provide detailed information on changes occurring within and after one outbreak because the averaged mononucleotide compositions for each month were analyzed. In fact, a horizontally long panel examining three human A subtypes (Fig. 5b) revealed that A% monotonically increased from 2009 to 2012 but it abruptly lost half of the increase in 2013 (arrowed), followed by a possible partial recursion increase in 2014; C% and G% (Supplementary Fig. 2a) also showed a monotonic decrease from 2009 to 2012, followed by an abrupt backset.

### Short oligonucleotide composition in influenza virus genomes

**Figure 6:**
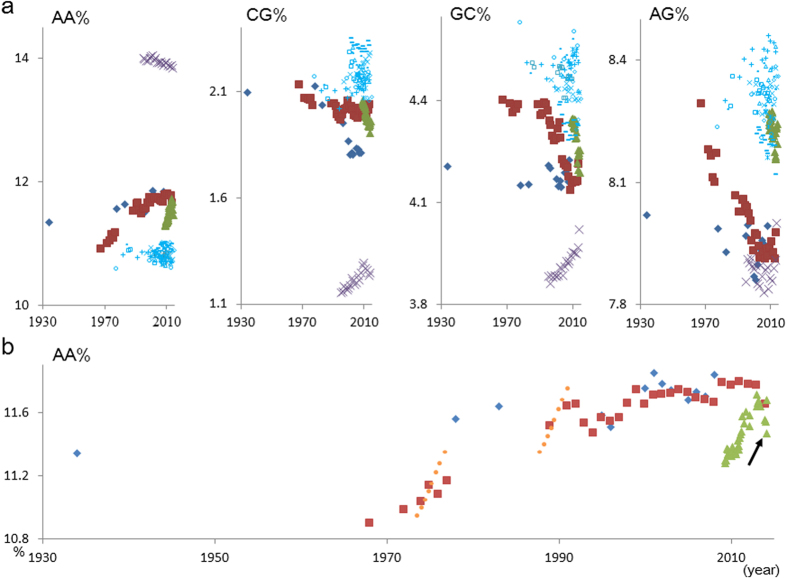
Time-series changes in dinucleotide compositions (%) for influenza viruses. (**a**) Dinucleotide compositions are plotted, as described in Fig. 5a. Correlation coefficient for human H1N1, H3N2 and pH1N1: 0.82, 0.94 and 0.87 for AA%, −0.81, −0.70 and −0.96 for CG%, −0.16, −0.84 and −0.90 for GC%, and −0.47, −0.93 and −0.66 for AG%, respectively. The null hypothesis was rejected for all four dinucleotides in three subtypes at the significance level of 0.01, except for GC% and AG% for H1N1. (**b**) A horizontally long panel for AA% of three human A subtypes is presented as described in Fig. 5b; Supplementary Fig. 2b also shows the abrupt backset in 2013 and 2014 for another dinucleotide, CG.

We next analyzed the di- to tetranucleotide compositions. Concerning correlation coefficients for the elapsed years in the case of H1N1 and H3N2 and for the elapsed months in the case of pH1N1, three subtypes show a common increasing or decreasing trend for thirteen of sixteen dinucleotides. The top four cases for the absolute value of averaged correlation coefficients for the three subtypes were examined in Fig. 6a. As observed for mononucleotides, all three subtypes appeared to move from avian A subtypes toward the human B type. A decrease in CG dinucleotides in A/U motifs during human-to-human transmission was previously reported^11^.

Detailed inspection of pH1N1 (Fig. 6b) showed a monotonic increase of AA% from 2009 to 2012 was followed by an abrupt backset in 2013 (arrowed). Here, we discuss the backset occurring after one outbreak may relate to the differential slopes of directional changes observed for different subtypes (Figs 5 and 6). pH1N1 strains, which are primarily derived from one outbreak, show an evidently steeper slope than H1N1 and H3N2 strains derived from multiple outbreaks, indicating a higher rate of pH1N1genome sequence changes. The first discussion was whether pH1N1 had an evidently higher mutation rate than H1N1 and H3N2. We propose here that without introducing the higher mutation rate of pH1N1, its seemingly higher rate of genome sequence changes can be explained by the model that conforms to the abrupt backset that occurred after an outbreak. When considering a viral infection spread, the ratio of persons with or without proper resistance to the viral infection (e.g., antibodies that are highly effective against the respective strain) becomes important. Therefore, the blooming strains (and their close relatives) in a certain outbreak (especially in a pandemic) may not be a very suitable strain for restarting the next outbreak because antibodies highly effective against the blooming strains are held by a large portion of humans. Less blooming strains, that are less adapted to humans, may become good starting strains for the next outbreak, resulting in a backset after one outbreak, as observed in Figs 5b and 6b and Supplementary Fig. 2. Hence, the evolutionary change focusing on strains belonging to a single outbreak looks higher than those belonging to multiple outbreaks, giving a steeper slope. To illustrate this interpretation, we added hypothetical data of H3N2 strains belonging to one outbreak as a series of small light-brown dots representing the dinucleotide compositions hypothesized for individual months, whose slope angle simply mimics that of pH1N1. Although other various mechanisms must be included to explain the backset after one outbreak, we propose that the steep slope of pH1N1 can be explained without introducing the higher mutation rate of pH1N1.

A wide range of tri- and tetranucleotides also showed decreasing or increasing trends common among the three subtypes (data not shown). The common increase or decrease of mono- and oligonucleotides should reflect the convergent-type evolution occurring in independently evolving viruses, which have invaded independently from nonhumans. We predict that the time-dependent changes required for efficient growth in human cells will repeat with a significant probability after future invasions from natural reservoir hosts and that this view is most likely applicable to a wide range of zoonotic RNA viruses.

### Long oligonucleotides in human influenza A genomes

Next, a time-series analysis was performed on long oligonucleotide occurrences. Long oligonucleotides are key elements of diagnostic RT-PCR primers^20^,^28^ and nucleic-acid therapeutics (e.g., siRNAs)^19^,^21^,^22^,^23^. When designing diagnostic and therapeutic oligonucleotides with long effectiveness for highly mutable viruses, we have to understand fundamental features of time-series directional changes of long oligonucleotides and to predict changes that will reoccur with a high probability. The sizes of PCR primers and therapeutic oligonucleotides range primarily from 15 to 30 nucleotides^19^,^21^,^22^,^23^. Thus, we analyzed 20-mer occurrences in genomes of three human influenza A subtypes, which independently invaded from nonhumans. Of a total of 4^20^ (*ca.* 1.1 trillion) types, *ca.* 1.4 million types of 20-mers were found in these genomes. Importantly, a time-series word count can analyze such big data without difficulty.

**Figure 7:**
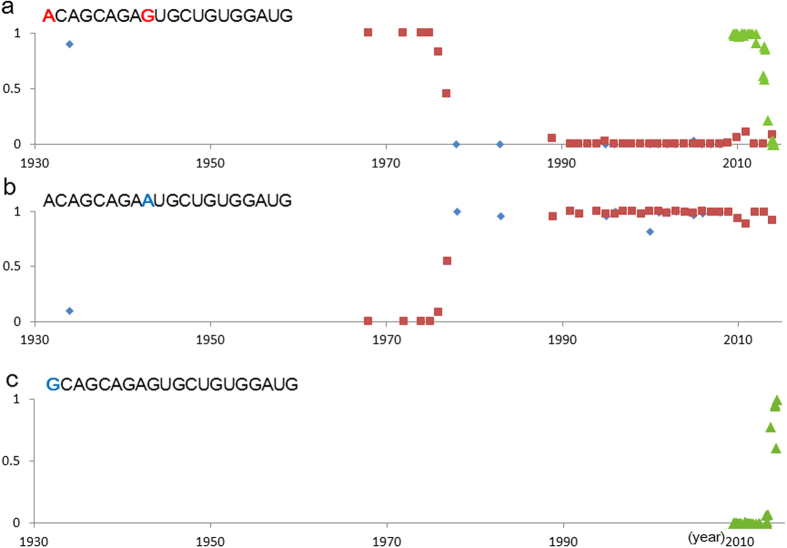
Time-series change in 20-mer occurrence per genome for influenza A viruses. (**a**) **A**CAGCAGA**G**UGCUGUGGAUG located in M2 cds region. (**b**) The 9-th G in the original 20-mer is changed to **A** (a nonsynonymous change, Ser−>Asn, AGU−>A**A**U) for H1N1 and H3N. **(c)** The first **A** in the original 20-mer is changed to **G** (a synonymous change for Glu, GA**A**−>GA**G**) for pH1N1.

After calculating correlation coefficients for each 20-mer for each subtype, we first selected all 20-mers having an evidently high or low correlation coefficient (>0.8 or <−0.8) in common for the three subtypes; all five 20-mers thus selected had negative correlation coefficients and were found to be mutually overlapped; i.e., these were components of one 24-mer. Figure 7a presents time-series occurrences for one 20-mer, ACAGCAGAGUGCUGUGGAUG; other four 20-mers gave practically the same decreasing pattern. At the beginning stage of three independent epidemics, most strains had one copy of the 20-mer, but during the course of human-to-human transmission the copy number decreased in the viral population and were almost completely lost at the final stage in all three subtypes; i.e., the strains having this 20-mer sequence progressively reduced their share in viral populations, regardless of differences in subtypes. We think that this common decrease reflects a certain inconvenience for efficient growth in human cells and will reoccur with a high probability after a future invasion. We next discuss this common decrease, in connection with siRNA designs.

Because of the social importance of influenza viruses, varieties of siRNAs have been designed as promising nucleic-acid therapeutics. Actually, VIRsiRNAdb^29^, which have complied the experimentally validated siRNA sequences, contains 96 siRNAs for influenza A viruses; and their length ranges from 19 to 21. When we searched viral siRNA sequences that were highly homologous to the 20-mers with an evident common increasing or decreasing trend with a BLAST search against VIRsiRNAdb, we found that four 19-mer influenza A siRNAs (virsi1635, 1640, 1645 and 1650), which had the same sequence (CAGCAGAGUGCUGUGGAUG) but were experimentally validated in different cell lines, showed a complete match to the 19-mer sequence harbored by the aforementioned five 20-mers, which are components of one 24-mer (AGGAACAGCAGAGUGCUGUGGAUG). Although these five 20-mers gave practically the same decreasing pattern to that listed in Fig. 7a, the compensatorily increased 20-mer responding to their decrease differed slightly in changed positions among the subtypes.

Figure 7b,c present patterns of the compensatorily increased 20-mers, which had nonsynonymous and synonymous changes from the original 20-mer, respectively (for details, see the Legend). This indicates that the sequence ranging over a certain length is possibly inconvenient for efficient growth in human cells ranges and that changes within the range can resolve the possible inconvenience. Since both synonymous and nonsynonymous changes were observed, the possible inconvenience should not relate to protein sequence. The information about the time-dependent directional change and the changed base should become important for designing siRNAs with long effectiveness.

### Future prospects

Finding time-series directional changes in long oligonucleotide occurrences for a large number of influenza A genomes can provide a new refinement strategy for designing therapeutic oligonucleotides and diagnostic RT-PCR primers with long effectiveness. Three influenza A subtypes may not be sufficient to judge the reoccurrence of the change, and therefore, our group has started to analyze the time-series changes in experimentally validated siRNA sequences, by focusing on both human and avian strains. An important issue is clarification of biological mechanisms responsible for directional changes in oligonucleotide occurrences after host changes. If the mechanism is clarified, reoccurrence in the future should become much clearly defined. Systematic analyses on a wide range of oligonucleotides with various lengths for the genomes of human and avian strains may give useful information about the possible mechanisms.

Judging from the directional changes observed in EBOV and pH1N1 outbreaks, genome sequences from strains isolated within several months after a new invasion appear to give reliable information about the directional change. If a large number of viral sequences becomes available, an averaging even per week for strains that are isolated in properly assigned geographic areas may clarify time-dependent directional changes. Undoubtedly, past sequences from the same or closely related viral genomes become important for comparison. In the present study, we focused on zoonotic RNA viruses whose reservoir hosts are vertebrates, but it is undoubtedly important to analyze other RNA viruses spread by bugs (e.g., ticks and mosquitos), which have caused serious emerging infectious diseases, such as Dengue fever and Zika virus disease^30^,^31^.

Results obtained with time-series word count are easily understandable even for those unfamiliar with evolutionary biology. When social importance of time-series word count of oligonucleotides becomes clear, experts in various fields will participate actively. Huge numbers of genome sequences, including those from disease-causing microbial strains, have accumulated rapidly because of revolutionary development in sequencing technologies and of social importance. In this era of big data accumulation, participation of experts in big data analysis becomes increasingly important for interdisciplinary efforts against big threats presented by infectious microorganisms.

## Methods

Genome sequences of human Zaire ebolavirus (EBOV) and MERS coronavirus strains were downloaded from the NCBI Virus Variation Database^24^ (http://www.ncbi.nlm.nih.gov/genome/viruses/variation/) on Dec. 20 and Dec. 31 (2015), respectively. Because the number of MERS virus sequences is small, compared with the other two viruses analyzed, a time-series analysis was conducted for the months having at least three strains, and when the number of strains for a certain month was less than 3, these were combined with those of the nearest neighboring month that had the lower number of strains than the other neighbor. From the NCBI Influenza Virus Resource^27^ (http://www.ncbi.nlm.nih.gov/genomes/FLU/), a total of *ca.* 200,000 segment sequences derived from *ca.* 25,000 influenza A and B virus strains were obtained on Sep. 1 (2015). We calculated mono- and oligonucleotide occurrences in eight genome segments of influenza virus strains and summed up the occurrences for each strain. To prevent possible misassignment from a large number of pH1N1 strains, relatively small numbers of human classical H1N1 strains isolated from 2009 were omitted from the present analysis. Influenza virus is a negative-sense single-stranded RNA virus, and therefore, the sequences analyzed are complementary to viral genome sequences. Computer codes are available from K.W. (k_wada@nagahama-i-bio.ac.jp).

## Additional Information

**How to cite this article**: Wada, Y. *et al.* Directional and reoccurring sequence change in zoonotic RNA virus genomes visualized by time-series word count. *Sci. Rep.* **6**, 36197; doi: 10.1038/srep36197 (2016).

**Publisher’s note:** Springer Nature remains neutral with regard to jurisdictional claims in published maps and institutional affiliations.

## Supplementary Material

Supplementary Information

## Figures and Tables

**Table 1:** Correlation coefficients for mono- and dinucleotide compositions.

	Guinea	Sierra Leone	Liberia
A	−0.68	**−0.93**	**−0.94**
C	0.73	0.78	**0.92**
G	−0.19	0.74	**0.87**
U	−0.40	−0.69	**−0.87**
AA	−0.26	**−0.87**	**−0.82**
AC	**0.83**	0.57	0.58
AG	−0.62	0.42	0.73
AU	**−0.82**	−0.61	−0.72
CA	0.35	0.47	0.60
CC	**0.80**	0.70	**0.84**
CG	−0.77	0.58	0.55
CU	**0.84**	0.23	0.74
GA	−0.69	0.19	0.44
GC	**−0.83**	0.78	0.73
GG	0.76	**0.80**	0.56
GU	**0.94**	0.39	0.68
UA	0.45	−0.33	−0.46
UC	0.47	−0.04	0.79
UG	**0.80**	0.33	0.63
UU	**−0.86**	**−0.91**	−0.77

Pearson’s correlation coefficients higher than 0.8 or less than −0.8 are bold. Statistical significance of the regression analysis not only of mono- and dinucleotide compositions listed here but also of other oligonucleotides for Ebolavirus, as well as for MERS and influenza viruses, are described in legends in Figs 1, 2, 3, 4, 5, 6.
