# Supplementary material for: Directional and reoccurring sequence change in zoonotic RNA virus genomes visualized by time-series word count
Source: Sci Rep. 2016 Nov 3;6:36197. doi: 10.1038/srep36197 (PMC5093548; doi:10.1038/srep36197)
Supplement: Supplementary Information [file srep36197-s1.pdf]

Directional and reoccurring sequence change in zoonotic RNA virus genomes visualized by time-series word count

Yoshiko Wada[1,2], Kennosuke Wada[1], Yuki Iwasaki[1], Shigehiko Kanaya[2] & Toshimichi Ikemura[1,*]

[1]Department of Bioscience, Nagahama Institute of Bio-Science and Technology, Tamura-cho 1266, Nagahama-shi, Shiga-ken 526-0829, Japan
[2]Nara Institute of Science and Technology, 8916-5 Takayama, Ikoma, Nara 630-0192 Japan

*Correspondence and requests should be addressed to T. I. (email: t_ikemura@nagahama-i-bio.ac.jp)

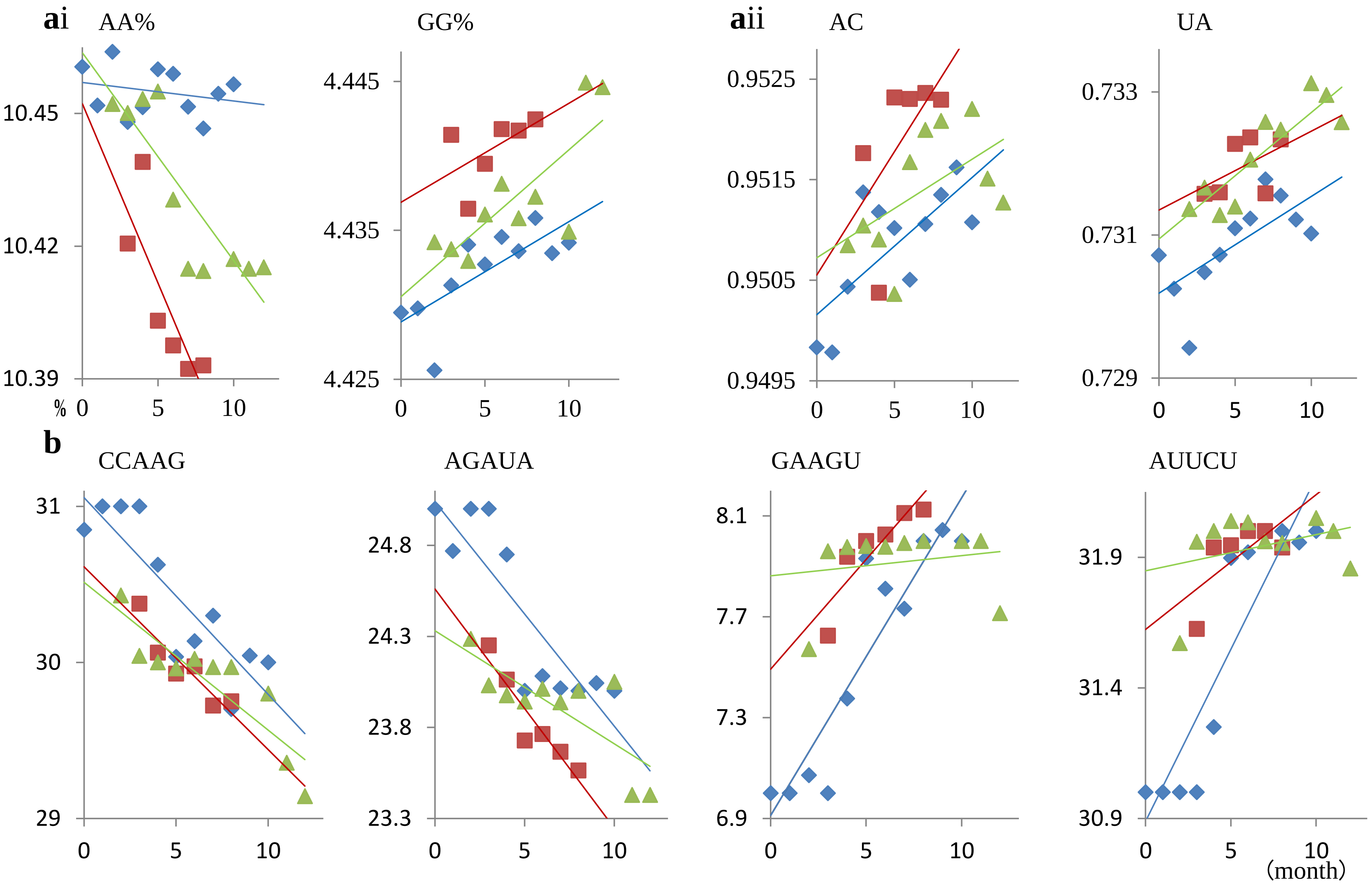

Supplementary Figure 1. Time-series change in di- and pentanucleotide occurrences for EBOV. (**a**) Averaged dinucleotide composition (i) and ratio of the observed to the expected occurrence (ii) for strains in each month, as well as regression lines, are presented as described in Fig. 2a and b, respectively. (**b**) Averaged 5-mer occurrence for EBOV strains in each month and regression lines are presented, as described in Fig. 3a.

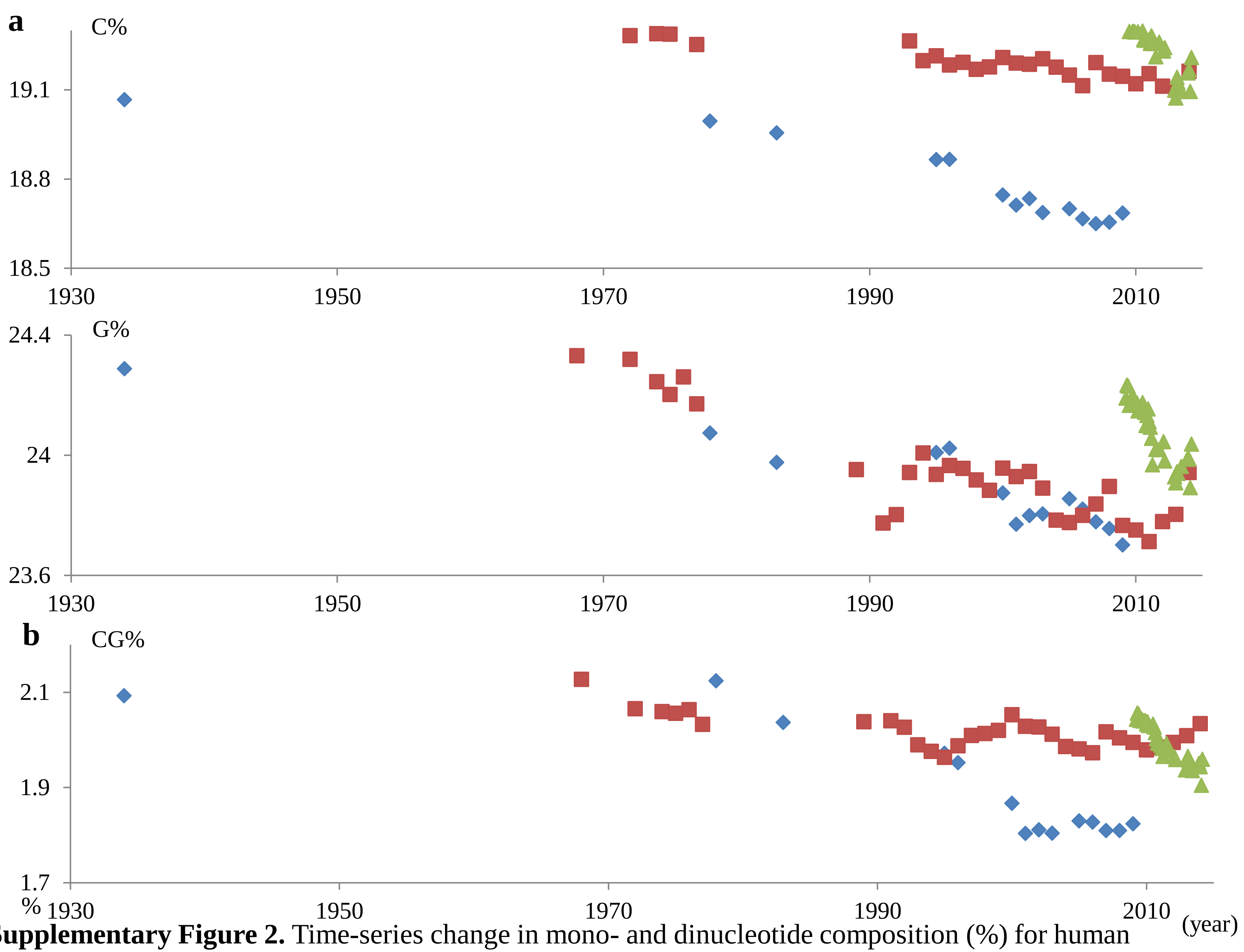

Supplementary Figure 2. Time-series change in mono- and dinucleotide composition (%) for human influenza A subtypes. Horizontally long panels for C% and G% (**a**) and CG% (**b**) are presented for clarifying the detailed changes occurring within and after one outbreak of pH1N1.
